# Supplementary material for: Prevalence and factors associated with adverse drug reactions among patients on highly active antiretroviral therapy at a tertiary hospital in south western Uganda: A cross-sectional study
Source: PLoS One. 2025 Apr 10;20(4):e0321015. doi: 10.1371/journal.pone.0321015 (PMC11984723; doi:10.1371/journal.pone.0321015)
Supplement: Attachment — Submitted filename: PONE-D-24-42042_reviewer (2).pdf [file pone.0321015.s003.pdf]

PLOS ONE

Prevalence and Factors Associated with Adverse Drug Reactions Among Patients on Highly Active Antiretroviral Therapy at a Tertiary Hospital in South Western Uganda: A cross-sectional study

--Manuscript Draft--

**Manuscript Number:**	PONE-D-24-42042
**Article Type:**	Research Article
**Full Title:**	Prevalence and Factors Associated with Adverse Drug Reactions Among Patients on Highly Active Antiretroviral Therapy at a Tertiary Hospital in South Western Uganda: A cross-sectional study
**Short Title:**	Adverse Drug Reactions Among Patients on Highly Active Antiretroviral Therapy
**Corresponding Author:**	Tadele Mekuriya Yadesa, Ph.D.<br>Kampala International University<br>Ishaka, UGANDA
**Keywords:**	Adverse drug reactions; associated factors; HAART; HIV
**Abstract:**	Background: HIV/AIDS remains a global health challenge, with significant prevalence in sub-Saharan Africa. Highly active antiretroviral therapy (HAART) is the mainstay treatment for HIV, and the number of people living with HIV (PLWHIV) on HAART has considerably increased worldwide. The use of HAART has led to improved patient outcomes; however, it is associated with adverse drug reactions (ADRs) and drug-drug interactions (DDIs), which pose serious concerns in the management of patients with HIV. The aim of the study was to determine the prevalence and factors associated with ADRs among patients on HAART.<br>Methodology: This was a hospital-based cross-sectional study carried out among 312 HIV patients on HAART attending HIV clinics at Mbarara Regional Hospital. Data was collected using an interviewer-administered, semi-structured questionnaire and a review of patient charts. ADRs were assessed for causality, severity, and preventability using standard tools. Logistic regression analysis was used to establish the association of variables.<br>Results: The prevalence of ADRs during this study was 76.0%. On assessment, 78.3% of the ADRs were mild and 76.6% of ADRs were definitely preventable. CD4 count below 200 cells/mm3 (AOR = 1.00, 95% CI: 1.00-1.02; p value = 0.04), primary education level (AOR = 3.27, 95% CI: 1.34-7.95; p value = 0.009), and secondary education level (AOR = 3.64, 95% CI: 1.39-9.52; p value = 0.009) were identified as independent risk factors. Patients who experienced a significant DDI were 5.66 times more likely to experience an ADR (p value = 0.02, 95% CI: 1.32-24.18).<br>Conclusion<br>There is a high prevalence of adverse drug reactions among patients with HIV on HAART. Low CD4 count and lower education levels are risk factors for ADRs in this population; therefore, targeted interventions should be developed for early identification and management. Significant drug-drug interactions are highly associated with the occurrence of ADRs among HIV patients on HAART, which calls for intensified pharmacovigilance and pharmaceutical care in this population.
**Order of Authors:**	Tadele Mekuriya Yadesa, Ph.D.
	Nangosya Moses
	Muyindike Rhoda Winnie
	Daniel Chans Mwandah
	Bontu Aschale Abebe
	Sarad Pawar Naik Bukke, PhD
	Narayana Goruntla
	Fredrick Atwiine
**Additional Information:**	

Powered by Editorial Manager® and ProduXion Manager® from Aries Systems Corporation

Question	Response
**Financial Disclosure**<br><br>Enter a financial disclosure statement that describes the sources of funding for the work included in this submission. Review the submission guidelines for detailed requirements. View published research articles from *PLOS ONE* for specific examples.<br><br>This statement is required for submission and **will appear in the published article** if the submission is accepted. Please make sure it is accurate.<br><br>**Funded studies**<br>Enter a statement with the following details:<br>• Initials of the authors who received each award<br>• Grant numbers awarded to each author<br>• The full name of each funder<br>• URL of each funder website<br>• Did the sponsors or funders play any role in the study design, data collection and analysis, decision to publish, or preparation of the manuscript?<br><br>Did you receive funding for this work?	The author(s) received no specific funding for this work.
**Competing Interests**<br><br>Use the instructions below to enter a competing interest statement for this submission. On behalf of all authors, disclose any competing interests that could be perceived to bias this work—acknowledging all financial support and any other relevant financial or non-financial competing interests.<br><br>This statement is required for submission and **will appear in the published article** if the submission is accepted. Please make sure it is accurate and that any funding sources listed in your Funding Information later in the submission form are also	The authors have declared that no competing interests exist.

declared in your Financial Disclosure statement.<br><br>View published research articles from *PLOS ONE* for specific examples.<br><br>**NO authors have competing interests**<br><br>Enter: *The authors have declared that no competing interests exist.*<br><br>**Authors with competing interests**<br><br>Enter competing interest details beginning with this statement:<br><br>*I have read the journal's policy and the authors of this manuscript have the following competing interests: [insert competing interests here]*<br><br>* typeset	
**Ethics Statement**<br><br>Enter an ethics statement for this submission. This statement is required if the study involved:<br><br>• Human participants<br>• Human specimens or tissue<br>• Vertebrate animals or cephalopods<br>• Vertebrate embryos or tissues<br>• Field research<br><br>Write "N/A" if the submission does not require an ethics statement.<br><br>General guidance is provided below. Consult the submission guidelines for detailed instructions. **Make sure that all information entered here is included in the Methods section of the manuscript.**	N/A

**Format for specific study types**<br><br>**Human Subject Research (involving human participants and/or tissue)**<br>• Give the name of the institutional review board or ethics committee that approved the study<br>• Include the approval number and/or a statement indicating approval of this research<br>• Indicate the form of consent obtained (written/oral) or the reason that consent was not obtained (e.g. the data were analyzed anonymously)<br><br>**Animal Research (involving vertebrate animals, embryos or tissues)**<br>• Provide the name of the Institutional Animal Care and Use Committee (IACUC) or other relevant ethics board that reviewed the study protocol, and indicate whether they approved this research or granted a formal waiver of ethical approval<br>• Include an approval number if one was obtained<br>• If the study involved *non-human primates*, add *additional details* about animal welfare and steps taken to ameliorate suffering<br>• If anesthesia, euthanasia, or any kind of animal sacrifice is part of the study, include briefly which substances and/or methods were applied<br><br>**Field Research**<br><br>Include the following details if this study involves the collection of plant, animal, or other materials from a natural setting:<br>• Field permit number<br>• Name of the institution or relevant body that granted permission	
**Data Availability**<br><br>Authors are required to make all data underlying the findings described fully available, without restriction, and from the time of publication. PLOS allows rare exceptions to address legal and ethical concerns. See the PLOS Data Policy and FAQ for detailed information.	Yes - all data are fully available without restriction

A Data Availability Statement describing where the data can be found is required at submission. Your answers to this question constitute the Data Availability Statement and **will be published in the article**, if accepted.<br><br>**Important:** Stating 'data available on request from the author' is not sufficient. If your data are only available upon request, select 'No' for the first question and explain your exceptional situation in the text box.<br><br>Do the authors confirm that all data underlying the findings described in their manuscript are fully available without restriction?	
**Describe where the data may be found in full sentences. If you are copying our sample text, replace any instances of XXX with the appropriate details.**<br><br>• If the data are **held or will be held in a public repository**, include URLs, accession numbers or DOIs. If this information will only be available after acceptance, indicate this by ticking the box below. For example: *All XXX files are available from the XXX database (accession number(s) XXX, XXX.).*<br>• If the data are all contained **within the manuscript and/or Supporting Information files**, enter the following: *All relevant data are within the manuscript and its Supporting Information files.*<br>• If neither of these applies but you are able to provide **details of access elsewhere**, with or without limitations, please do so. For example:<br><br>*Data cannot be shared publicly because of [XXX]. Data are available from the XXX Institutional Data Access / Ethics Committee (contact via XXX) for researchers who meet the criteria for access to confidential data.*<br><br>*The data underlying the results presented in the study are available from (include the name of the third party*	All relevant data are within the manuscript and its Supporting Information files.

and contact information or URL).<br>• This text is appropriate if the data are owned by a third party and authors do not have permission to share the data.<br><br>* typeset	
Additional data availability information:	

Powered by Editorial Manager® and ProduXion Manager® from Aries Systems Corporation

Manuscript

Click here to access/download;Manuscript;Manuscript Revised.docx

Prevalence and Factors Associated with Adverse Drug Reactions Among Patients on Highly Active Antiretroviral Therapy at a Tertiary Hospital in South Western Uganda: A cross-sectional study

Nangosya Moses[1], Muyindike Rhoda Winnie[2], Daniel Chans Mwandah[3,4], Bontu Aschale Abebe[5], Sarad Pawar Naik Bukke[6], Narayana Goruntla[7], Fredrick Atwiine[1], Tadele Mekuriya Yadesa[1,7]*

Author Affiliations

[1] Department of Pharmacy, Mbarara University of Science and Technology, Mbarara, Uganda

[2] Department of Internal Medicine, Mbarara University of Science and Technology, Mbarara, Uganda

[3] Department of Pharmacology and Therapeutics, Mbarara University of Science and Technology, Mbarara, Uganda

[4] Department of Pharmacology and Toxicology, School of Pharmacy, Kampala International University, Ishaka, Uganda

[5] School of Nursing, Kampala International University, Ishaka, Uganda

[6] Department of Pharmaceutics and Pharmaceutical Technology, School of Pharmacy, Kampala International University, Ishaka, Uganda

[7] Department of Clinical Pharmacy and Pharmacy Practice, School of Pharmacy, Kampala International University, Ishaka, Uganda

***Corresponding Author:** *Tadele Mekuriya Yadesa. Department of Clinical Pharmacy and Pharmacy Practice, School of Pharmacy, Kampala International University, P.O.Box 20000, Ishaka, Uganda. Email: maatiikoo4@gmail.com*

Abstract

Background: HIV/AIDS remains a global health challenge, with significant prevalence in sub-Saharan Africa. Highly active antiretroviral therapy (HAART) is the mainstay treatment for HIV, and the number of people living with HIV (PLWHIV) on HAART has considerably increased worldwide. The use of HAART has led to improved patient outcomes; however, it is associated with adverse drug reactions (ADRs) and drug-drug interactions (DDIs), which pose serious concerns in the management of patients with HIV. The aim of the study was to determine the prevalence and factors associated with ADRs among patients on HAART.

Methodology: This was a hospital-based cross-sectional study carried out among 312 HIV patients on HAART attending HIV clinics at Mbarara Regional Hospital. Data was collected using an interviewer-administered, semi-structured questionnaire and a review of patient charts. ADRs were assessed for causality, severity, and preventability using standard tools. Logistic regression analysis was used to establish the association of variables.

Results: The prevalence of ADRs during this study was 76.0%. On assessment, 78.3% of the ADRs were mild and 76.6% of ADRs were definitely preventable. CD4 count below 200 cells/mm^3 (AOR = 1.00, 95% CI: 1.00-1.02; *p* value = 0.04), primary education level (AOR = 3.27, 95% CI: 1.34-7.95; *p* value = 0.009), and secondary education level (AOR = 3.64, 95% CI: 1.39-9.52; *p* value = 0.009) were identified as independent risk factors. Patients who experienced a significant DDI were 5.66 times more likely to experience an ADR (*p* value = 0.02, 95% CI: 1.32-24.18).

Conclusion

There is a high prevalence of adverse drug reactions among patients with HIV on HAART. Low CD4 count and lower education levels are risk factors for ADRs in this population; therefore, targeted interventions should be developed for early identification and management.

Significant drug-drug interactions are highly associated with the occurrence of ADRs among HIV patients on HAART, which calls for intensified pharmacovigilance and pharmaceutical care in this population.

Keywords: Adverse drug reactions, associated factors, HAART, HIV

Background

Globally, about 39.9 million persons live with HIV (1), of which 30.7 million people had access to antiretroviral therapy (ART) by the end of 2023 (2). Sub-Saharan Africa (SSA) accounts for about 67% of all people living with HIV worldwide (3). Uganda, which is one of the ten high-burden countries in SSA, had a nationwide HIV prevalence of 5.1% among adults aged 15-49 years in 2023 (3), with the Mbarara district having a prevalence of 13.1% (4, 5). The number of people gaining access to antiretroviral therapy (ART) globally with the use of highly active antiretroviral therapy (HAART), which involves the use of a combination of three or more antiretroviral drugs to treat HIV infection (6), with the aim of suppressing viral replication, restoring the immune system response, stopping disease progression, increasing survival rates, reducing morbidity, and improving quality of life (7). The use of HAART has led to a remarkable decrease in AIDS-related deaths by 51% from about 1.3 million beings in 2010 to 630,000 in 2023 (2, 8).

Despite the effectiveness of HAART in managing HIV, there is a high risk of adverse drug reactions (ADRs) associated with HAART due to the complexity, toxicity, and potential for drug-drug interactions (9, 10). Additionally, these patients tend to have comorbid conditions that also require drug therapy (11), which predisposes these patients to ADRs from the associated use of drugs. An adverse drug reaction can be defined as a harmful or unpleasant reaction that is due to an intervention that is related to the use of a medicinal product that predicts hazard from future administration and hence warrants prevention, treatment, or

adjustment in dosage regimen, or discontinuation of the product (12, 13). ADRs negatively impact patients' quality of life, lead to treatment discontinuation, medication non-adherence, and poor patient outcomes (14-16).

Healthcare professionals play a key role in preventing, identifying, and managing ADRs by closely monitoring patients, promoting medication safety practices, and providing appropriate education and counseling regarding potential risks and side effects (17-19). Pharmacists in particular can offer comprehensive strategies that focus on medication reconciliation, proper dosing, and patient education, which help minimize ADRs and improve patient outcomes (20, 21). Several studies have been carried out in patient populations on specific HIV treatment regimens with varying prevalence and factors leading to ADR occurrence in this population in both time and location (22-24).

Given the benefits of HAART in managing HIV and the potential impact of ADRs on patient outcomes, it is key to understand the prevalence and factors associating with ADR occurrence in patients on HAART. However, currently there is a lack of comprehensive research and evidence-based results regarding the prevalence and factors contributing to ADRs, especially among HIV patients on HAART in our setting. The study therefore aimed to determine the prevalence and factors associated with ADR occurrence among patients on HAART at a tertiary hospital in Uganda.

Methods

Study Setting and Period.

The Mbarara Regional Referral Hospital's (MRRH) HIV/Immune Suppression Clinic (ISS), located 280 km from Kampala, served as the study's location. MRRH is a 350-bed regional referral hospital located in southern Uganda. The hospital is currently serving a population of about four million people in the catchment area of the districts of Kiruhura, Mbarara, Sheema,

Bushenyi, Rwampara, Ibanda, Buhweju, Rubirizi, Mitooma, Isingiro, Lyantonde, Rakai, Ntungamo, and other neighbouring districts. The HIV clinic has a total of 11,218 active clients on ART and receives about 80 to 150 clients per day. A physician leads the clinic as the in-charge, supported by a paediatrician, medical officers, a pharmacist, 5 registered nurses, 8 enrolled nurses, 2 dispensers, 4 laboratory technicians, 4 counsellors, a biostatistician, 4 data clerks, and other support staff. The clinic has a separate laboratory embedded within it for common screening tests, as well as some routine follow-up and monitoring parameters. We conducted the study between 11th September 2023 and 31st of October 2023.

Study Design

This was a prospective cross-sectional study conducted among HIV patients actively on HAART who attended ISS clinic of the Mbarara Regional Referral Hospital.

Study Population

The study included all adult PLW HIV (18 years and older) who were on HAART at the ISS Clinic of MRRH during the study period, actively on follow-up, and willing to participate. We excluded patients who started HAART less than a month ago and those with incomplete records in their files (HAART regimen, start date, weight) from this study.

Sample Size Determination.

The sample size of this study was estimated using Fisher's formula for estimating sample size, $\mathbf{n = Z^2\alpha/_2\, P(1\text{-}P)/\delta^2}$ (25). The prevalence of ART-related ADRs of 24.4% was obtained from a systemic review study in Africa (26). With the addition of 10% to compensate for incomplete data, the sample size was determined to be **312 patients.**

Sampling Technique

At the HIV clinic, we assigned consecutive numbers to patients' files every day of the study period, starting from one, based on their follow-up schedules. We generated random numbers using MS-Excel version 2016. Every patient at the clinic received an equal chance of selection.

The same procedure continued on clinic days until a number of 312 was achieved, which was our sample size.

Data collection tool and procedure

We obtained informed consent from each patient to participate in the study. We used a data abstraction form to extract data from patients' medical charts, including diagnostic data. We conducted interviews with patients using a structured interviewer-administered questionnaire to gather data not available in the patient files. We evaluated the known adverse reaction profile of each drug using Ugandan Clinical Guidelines 2023 (27), British National Formulary (BNP) version 85, and Up-To-Date. We used the Naranjo ADR assessment scale (28) to rate the casual relationship between an ADR and the suspected medication. We excluded all doubtful ADRs and only considered those rated as possible, probable, or definite for discussion and verification. We established the severity of ADRs by applying the modified Hartwig and Siegel criteria, which consisted of seven items and three severity categories: "mild, moderate, and severe ADRs" (29). ADRs were further assessed for preventability by using the 9-item Schumock and Thornton criteria, categorizing adverse drug reactions into definitely preventable, probably preventable, or not preventable (30). Lexicomp® Drug Interaction Checker software was utilized to identify clinically significant drug-drug interactions (DDIs) and classified them according to risk rating: A (no known interaction), B (no action needed), C (monitor therapy), D (consider therapy modification), and X (avoid combination).

Research assistants followed the Ugandan Ministry of Health's Covid-19 and Ebola Protocols. These included use of personal protective equipment like double masking, sanitizing before, during, and after handling patient files, disinfecting the area, and hand washing with soap and water.

Data Quality Control

We recruited and trained three research assistants on the data collection procedures. We pretested the data collection tool and its reproducibility with 20 participants to check for any ambiguity and appropriateness. Good clinical practice guidelines were followed. We used a well-defined data collection procedure. The principal investigator closely supervised the research assistants and provided timely revisions about data completeness and appropriateness.

Data Analysis

Data was checked for completeness, entered into Microsoft Excel 2016 for data cleaning and then exported to Stata version 13 for analysis. The prevalence of ADRs and potential DDI was analyzed using descriptive statistics and presented in measures of central tendency, frequencies, and percentages. We performed bivariate and multivariate logistic regression to identify the factors associated with ADRs. The multivariate logistic regression included variables that had a p-value of less than 0.25 in the bivariate logistic regression. A p-value of <0.05 and a 95% confidence interval were used as cutoff points for determining the statistical significance of associations.

Ethical Approval and consent to participate

This study was conducted in accordance with the Declaration of Helsinki. The study was approved by the Mbarara University of Science and Technology, Research and Ethics Committee (Reference Number: MUST-2023-1074). Clearance to carry out the study was obtained from the Mbarara Regional Referral Hospital. As all the participants were 18 years of age or older, we obtained written informed consent directly from each participant before recruiting in this study. Patients were given a choice between English or Runyankure versions of the approved consent forms. For patients who could not read, the patient's caretaker or any other person approved by the patient read the consent to the patient and served as a witness.

Results

Socio-demographic characteristics of participants

A total of 312 patients were interviewed and their records reviewed. The majority of the participants were within the age range of 45-55 years (33.0%). There were more females (60.0%) than males (39.1%). The age range was 23–80 years with a mean of 45.2 (SD, 10.6). The majority of them were married (58.7%), those who had primary education were 49.4%, peasants were 36% and 53.5% live in an urban area (***Table 1***).

Table 1: Socio-demographic characteristics of participants Patients with HIV on HAART at Mbarara Regional Referral Hospital

Variable	Category	Frequency n=312	Percentage
**Age group (years)**	<35	54	17.3
	35-<45	91	29.2
	45-<55	103	33.0
	≥55	64	20.5
**Sex**	Male	122	39.1
	Female	190	60.9
**Marital Status**	Single	18	5.8
	Married	183	58.7
	Divorced	51	16.4
	Widowed	60	19.2
**Educational attainment**	None	34	10.9

	Primary	154	49.4
	Secondary	90	28.9
	Tertiary	34	10.9
**Occupational status**	Unemployed	9	2.9
	Employed	37	11.9
	Peasant	112	36.0
	Business	82	26.3
	Others	72	23.1
**Place of residence**	Rural	90	28.9
	Semi urban	55	17.6
	Urban	167	53.5
**Alcohol Use**	Yes	98	31.4
	No	214	68.6
**Smoking**	Yes	28	9.0
	No	383	91

Clinical characteristics at the beginning of ART

Among the selected patients, more than 99% had started HAART at WHO stage 1. More than 50% of patients started HAART with CD4 cells with more than 200 cells/mm^3 CD4 count (***Table 2***). 30.8% of the patients had other comorbidities, with hypertension being the most common (***Figure 1***).

Table 2: Participants clinical characteristics patients with HIV on HAART at MRRH

**Variable**	**Category**	**Frequency**	**Percentage**
Baseline CD4 count	>200	189	61.6
	<200	118	38.4
Current CD4 count	>200	256	83.9
	<200	49	16.1
Comorbidity	Yes	93	30.8
	No	209	69.2
Opportunistic Infection	Yes	118	38.2
	No	191	61.8
Medication Use Counselling	Yes	311	99.7
	No	1	0.3

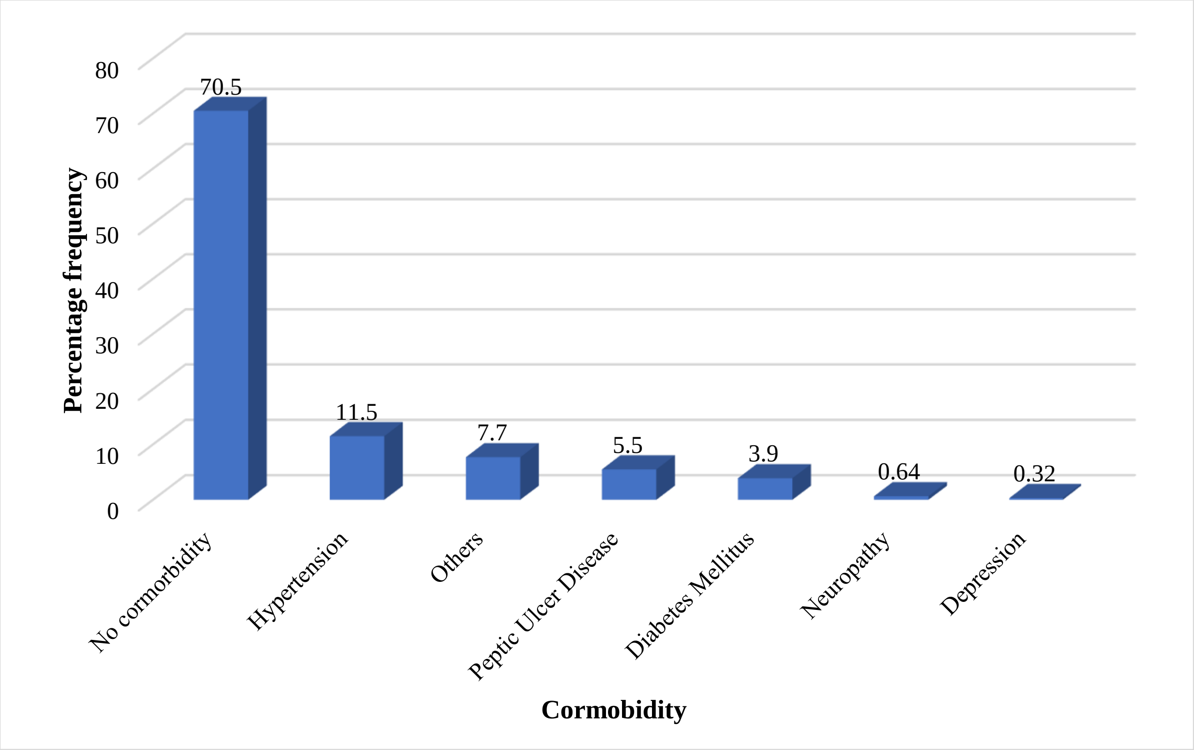

Figure 1: Comorbidity status of patients with HIV on HAART at MRRH

HAART drug regimens

The initial regimen was TDF/3TC/EFV for 153 (49.4%), TDF/3TC/DTG for 45 (14.1%), AZT/3TC/NVP for 50 (16.0%), AZT/3TC/EFV for 21 (6.7%), TDF/3TC/LPV/r for 12 (3.9%), TDF/3TC/ATV/r for 9 (2.9%) and other ART 14 (4.5%) of patients. The majority of the patients were currently on TDF/3TC/DTG 292 (92.3%), followed by TDF/3TC/EFV (2.6%) and 1.9% were on ABC/3TC/DTG (***Table 3***). About 185 (59.5%) patients reported using other medicines other than for HIV treatment. From those who took other drugs other than ART, 16 (5.1%) patients were on co-trimoxazole and 305 (98.1%) patients had ever been on isoniazid prophylaxis. About 75 (24.7%) of the patients reported using herbal drugs.

Table 3: Current HAART regimen among Patients with HIV at MRRH

HAART Regimen	Frequency	Percentage
TDF/3TC/DTG	292	92.3
TDF/3TC/EFV	8	2.6
ABC/3TC/DTG	6	1.9
AZT/3TC/DTG	1	0.3
AZT/3TC/NVP	1	0.3
TDF/3TC/ATZ/r	2	0.64
TDF/3TC/LPV/r	2	0.64

Prevalence of ADR

The number of patients who reported at least one ADR was 239 out of 312, representing 76.6% (***Error! Reference source not found.***).

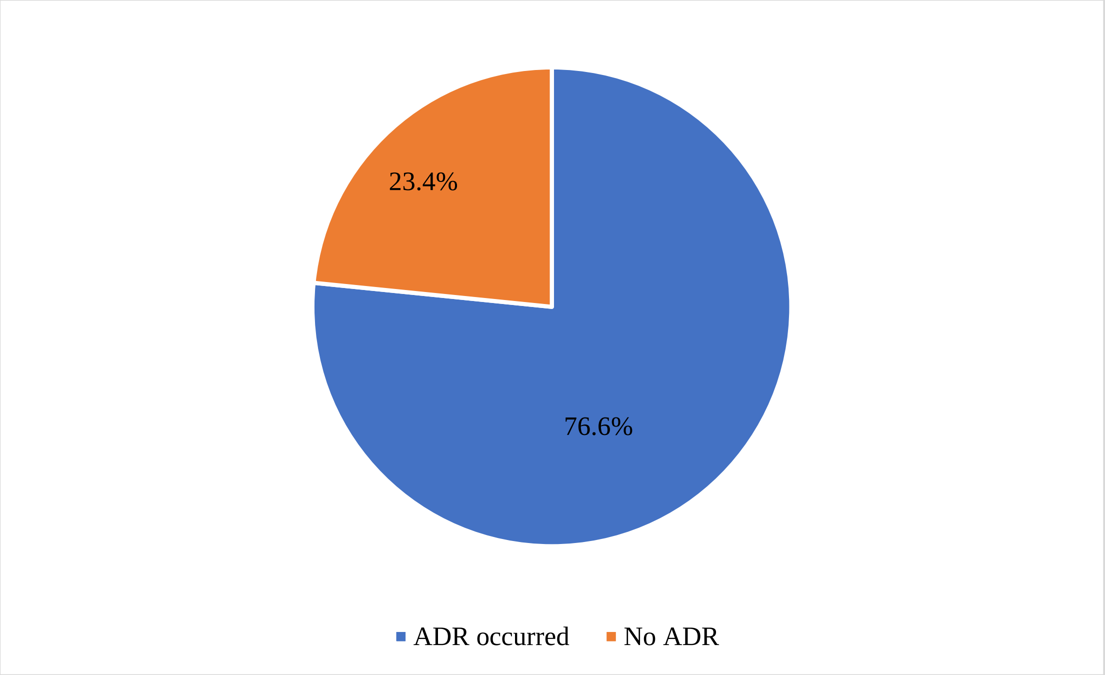

Figure 2: Prevalence of ADRs among patients living with HIV at MRRH

ADR Assessment, severity and Preventability

Using the Naranjo ADR causality rating scale, 60.7% of ADRs were rated as probable, 39% as possible and 0.3% rated as definite. Modified Hartwig and Siegel's scale was used for severity assessment, where 78.3% of the ADRs were mild, 21.4% moderate and 0.3% severe. Preventability assessment was done by using the Schumock and Thornton scale and it was found that 76.6% of the ADRs were definitely preventable, 11.0% were probably preventable, and 12.4% were non-preventable (***Figure 3***).

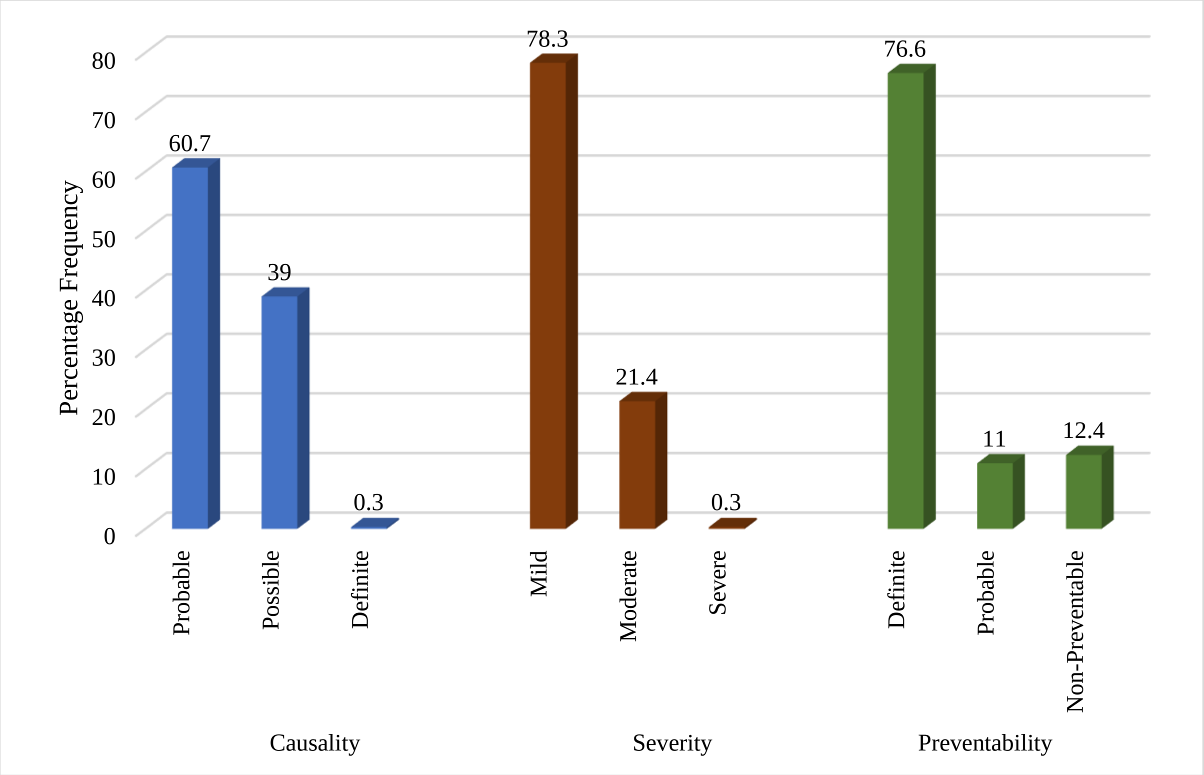

Figure 3: ADR assessment, severity and preventability among patients with HIV on HAART at MRRH

Out of the 76% ADRs in HAART detected, TDF was the most implicated with 141 (47%), DTG with 78 (26%), 3TC 71 (23.8%) and others 10 (3.2%) of the ADRs. The most commonly reported adverse drug reaction was low back pain (17.2%), difficulty falling asleep (9.9%), fatigue 66 (9.7%), skin manifestations (9.3%) and GIT manifestations (8.2%). Common actions

taken by those reported were counselling about the drug and encouraging them to continue with the drugs or withholding the drugs (***Table 4***).

Table 4: Commonly Reported ADRs among patients with HIV on HAART at MRRH

ADR	Percentage Frequency
Low back pain	17.2
Difficulty falling asleep	9.9
Fatigue	9.7
Skin manifestations (Rashes & itching)	9.3
GIT manifestations (Nausea, Vomiting and diarrhea)	8.2
Abdominal pain	8.1
Drowsiness/Dizziness	6.3
Excessive Eating	4.4
Low appetite	4
Excessive drinking	3.8
Headache	2.5
Numbness of the extremities	2.4
Renal problems	1.2
Others	9.2

Factors associated with adverse drug reactions.

Variables with *p*-value less than 0.25 at bivariate analysis included polypharmacy, age, gender, education level, baseline CD4 count, current CD4 count, comorbidity, change of regimen, over-the-counter medicine use (OTC), and herbal drug use. These variables were included in multivariable logistic regression and only current CD4 count below 200 cells/mm^3 (AOR = 1.88, 95% CI: 1.23-1.99; *p* value = 0.041) vs ≥200 cells/mm^3, and primary level of education (AOR = 3.27, 95% CI: 1.34-7.95; *p* value = 0.009) and secondary level of education (AOR = 3.64, 95% CI: 1.39-9.52; *p* value = 0.007) vs tertiary educational level, were significantly associated with occurrence of at least one ADR **(Table 5)**.

Table 5: Univariate and multivariate logistic regression of the factors associated with ADR among patients living with HIV at MRRH

**Variables**	**Category**	**COR (95 CI)**	**P value**	**AOR (95 CI)**	**P value**
Gender	Male	1			
	Female	1.29 (0.76-2.20)	0.910		
Age (years)	< 35	1			
	35-55	0.98 (0.45-2.10)	0.953		
	>55	1.69 (0.69-4.11)	0.250		
Polypharmacy	No	1		1	
	Yes	2.22(1.07-4.61)	0.032	1.40(0.98-1.88)	0.241
Education	Tertiary	1		1	
	Primary	3.26(1.49-7.16)	0.003	3.27(1.34-7.95)	**0.009**

	Secondary	3.65(1.54-8.68)	0.003	3.64(1.39-9.52)	**0.007**
Comorbidity	No	1		1	
	Yes	2.36(1.22- 4.57)	0.011	1.55(0.13- 1.04)	0.098
Current CD4	> 200	1		1	
	< 200	4.21 (2.34-8.01)	0.008	1.88(1.23-1.99)	**0.041**
Baseline CD4	> 200	1		1	
	< 200	1.12 (1.01-1.82)	0.029	1.01(0.87-1.56)	0.834
HAART regimen change	No	1		1	
	Yes	1.83(0.86-3.89)	0.116	1.14 (081-2.11)	0.412
OTC use in the previous 4 weeks	No	1		1	
	Yes	1.72 (1.01-2.92)	0.044	1.32 (0.47-2.89)	0.651
Herbal use in the previous 4 weeks	No	1		1	
	Yes	1.62(0.83-3.15)	0.158	1.03 (0.39-2.40)	0514

Discussion

In this study, the prevalence of ADR occurrence among HIV patients on HAART was determined to be 76.6%. The prevalence is considerably higher than the one reported from pooled studies in Africa, which gave a prevalence of 28% (26), and also higher than 7.9% reported in Nigeria (31), 25.5% in Ethiopia (32), 13.8% in India (33) and 9.4% reported in Ghana (34). The possible explanation could be due to differences in the pharmacovigilance practices in different settings, the study employed in some studies, the number of participants in the study and the duration of follow-up period of different studies. The higher prevalence of ADRs in this study can also be explained by the combined use of patient interviews and reviews

of patient charts that might have led to higher ADR detection. Given the ever-changing HIV treatment regimen (35, 36), the difference could be as a result of the difference in regimen studied in different studies, as ADR occurrence has shown to differ based on the toxicity of the regimen used (37, 38). However, the reported prevalence in this study is lower than 85.5% reported in Brazil (39) and 94% from Iran (40). Possible reasons for a lower prevalence could be the cross-sectional design in the current study, which typically captures data at a single point in time, while prospective studies, like the study from Brazil, are generally more robust in terms of data collection and follow-up, which may lead to a more comprehensive assessment of ADRs.

In our study, back pain, skin manifestations including rash and itching, and GIT manifestations were the most reported ADRs. Back pain due to antiretroviral drugs has been linked to neuropathy caused by some of these drugs, although HIV as a chronic illness has also may cause back pain due to increased inflammation, exaggerated immune system responses, and nerve damage, co-existing conditions (41, 42). Some drugs, such as Non-Nucleoside Reverse Transcriptase Inhibitors such as efavirenz, have shown to cause rash and itching and the profound increase in CD4 counts during the initial phase of treatment has been proposed as a possible explanation for the development of the drug-induced skin rash (43). The gastrointestinal symptoms, including diarrhea, nausea, vomiting, and dyspepsia, is caused by GI intolerance by most ARV drug combinations, which usually appears during the first weeks of therapy, and usually mild and transient in most patients (44).

The severity of ADRs reported in this study was mild in 78.3% of all cases. This could be explained partly by the efforts to improve pharmacovigilance and management of side effects in HIV clinics (45-47), most participants did not have any comorbidities, which also increase the severity of ADRs (48, 49). The current drug regimen that most of the participants were taking also included newer ARVs such as dolutegravir-based regimens that have a better

toxicity profile, which could explain the mild cases observed (50, 51). This study also found out that about 76.6% of all ADR cases reported were preventable. These findings underscore the importance of pharmaceutical care and therapeutic drug monitoring, including avoiding drugs that had previously caused an ADR, avoiding inappropriate medications, optimizing the dosage regimen, conducting the necessary monitoring tests regularly, and checking for significant drug interactions, all of which have been shown to prevent the occurrence of ADRs among HIV patients on HAART (13, 52, 53).

Participant’s education level and current CD4 count were identified as independent risk factors of ADR occurrence among HIV patients in this study. Participants who had primary and secondary education had about 3.27 and 3.64 odds of developing an ADR respectively, as compared to patients who attended tertiary education. These findings are comparable to those reported in Ethiopia (54), Northern Nigeria (55), and India (56). This could be explained by the fact that patients with higher education may understand better drug instructions, adherence, and good nutritional status and promote better self-care and monitoring (57). Low CD4 count was significantly associated with the occurrence of ADRs, as most patients that developed ADRs had CD4<200 cells/mm3. This is in line with several studies from India (58), Brazil (39) and Ethiopia (59). Increased risk soon after HAART initiation is thought to result from the rapid rise of CD4 lymphocytes, which can be an important stimulus of ADRs as part of an immune reconstitution inflammatory syndrome (60). Immunodeficiency can lead to dysregulation in drug metabolism, hence potentially affecting the pharmacokinetics of antiretroviral drugs and increasing the risk of ADRs (61).

In this study, the patients with significant DDIs were 5.66 times more likely to develop an ADR and this association was statistically significant. DDIs are more prevalent in HIV patients, and they are important causes of preventable ADRs, as they are at an increased risk of multimorbidity and polypharmacy (62). DDIs are usually predictable and manageable; there

ADRs caused by DDIs may be prevented by monitoring the patient closely or replacing the responsible drugs with other medications in case a DDI is suspected (63). It is important that healthcare providers regularly conduct comprehensive medication reviews to recognize potentially interacting drugs and intervene in time.

Limitations of the study

The study's reliance on a single-centre setting could affect the external validity of the study. Secondly, the fact that ADRs were assessed based on patient self-reporting, which might introduce recall bias or underreporting. The study's duration may not capture long-term ADR patterns and the evolving nature of patient responses to HAART.

Conclusion

The study revealed a high prevalence of ADRs (76.6) among HIV patients on HAART at Mbarara Regional Referral Hospital, emphasizing the need for vigilant monitoring and management strategies within HIV care. Education level and current CD4 count demonstrated a significant association with the development of ADRs, highlighting the importance of tailored interventions for these subgroups. The study identified a statistically significant association between clinically significant DDIs and the occurrence of ADRs, underscoring the importance of pharmaceutical care for prompt identification of drug interactions and ADR management in HIV patient care. Future researchers should consider multi-centered and longitudinal studies to generate more precise and generalizable results.

Abbreviations

ABC: Abacavir; **ADEs:** Adverse Drug Events; **ADRs:** Adverse drug reactions; **AIDS**; Acquired Immunodeficiency Syndrome; **BNF**: British National Formulary; **ART**: Antiretroviral therapy; **ARVs**: Antiretrovirals; **ATV/r**: Atazanavir/ritonavir; **AZT** Zidovudine; **CD4:** Clusters of differentiation; ***COR:*** Crude odds ratios*;* **DTG**: Dolutegravir;

EFV: Efavirenz; **HAART**: Highly Active Anti-Retroviral Treatment; **HIV**: Human immuno deficiency virus; **EMTCT:** Eliminating mother to child transmission of HIV; **IRB**: Institutional Review Board; **ISS**: Immune Suppression Syndrome; **LPV/r**: Lopinavir/ritonavir; **MoH**: Ministry of Health; **MRRH**: Mbarara Regional Referral Hospital; **MUST**: Mbarara University of Science and Technology; **NVP:** Nevirapine; **OTC** Over the counter; **PLWHA**: People Living with HIV/AIDS; **REC**: Research Ethics Committee; **TAF**: Tenofovir alafenamide; **TDF:** Tenofovir; **3TC:** Lamivudine; **SSA** Sub Saharan Africa; **UNAIDS**: United Nations Programme on HIV/AIDS; **WHO:** World Health Organization

Declarations

Ethical Approval and consent to participate

This study was conducted in accordance with the Declaration of Helsinki. The study was approved by the Mbarara University of Science and Technology, Research and Ethics Committee (Reference Number: MUST-2023-1074). Clearance to carry out the study was obtained from the Mbarara Regional Referral Hospital. Informed consent was sought from eligible participants before their recruitments.

Consent for publication

All authors agreed to the submission of this manuscript for publication

Availability of data and materials

The datasets used and analyzed during this study shall be availed by the author on request.

Competing interests

The authors declare that they have no competing interests.

Funding

This study was entirely funded by the author and did not receive any external funding

Authors' contributions

All authors made a significant contribution this study during conceptualization and study design, data collection, data analysis, manuscript writing and review. All authors agree to be accountable for all aspects of this work.

Acknowledgements

We would like to thank all the staff of ISS clinic of MRRH for their collaboration and support during the data collection. We would also like to extend our thanks to the participants for consenting to take part in this study.

References

1. WHO. HIV Global situation and trends: World Health Organization; 2024 [Available from: https://www.who.int/data/gho/data/themes/hiv-aids#:~:text=Globally%.

2. UNAIDS. Global HIV & AIDS statistics — Fact sheet: UNAIDS; 2024 [Available from: https://www.unaids.org/en/resources/fact-sheet#:~:text=Global%20HIV%.

3. Moyo E, Moyo P, Murewanhema G, Mhango M, Chitungo I, Dzinamarira T. Key populations and Sub-Saharan Africa's HIV response. Front Public Health. 2023;11:1079990.

4. Musana H, Ssensamba JT, Nakafeero M, Mugerwa H, Kiweewa FM, Serwadda D, et al. Predictors of failure on second-line antiretroviral therapy with protease inhibitor mutations in Uganda. AIDS Research and Therapy. 2021;18(1):1-10.

5. Betunga B, Atuhaire P, Nakasiita C, Kanyamuneza C, Namiiro P, Tugume J, et al. Factors influencing the use of multiple HIV prevention services among transport workers in a city in southwestern Uganda. PLOS Global Public Health. 2023;3(3):e0001350.

6. Ginat DT, Small JE, Schaefer PW. Neuroimaging Pharmacopoeia: Springer Nature; 2022.

7. Günthard HF, Saag MS, Benson CA, Del Rio C, Eron JJ, Gallant JE, et al. Antiretroviral drugs for treatment and prevention of HIV infection in adults: 2016 recommendations of the International Antiviral Society–USA panel. Jama. 2016;316(2):191-210.

8. Zhao Y, Wei L, Dou Z, Zhao D, Gan X, Wu Y, et al. Changing Mortality and Patterns of Death Causes in HIV Infected Patients - China, 2013-2022. China CDC Wkly. 2023;5(48):1073-8.

9. Dionne B. Key principles of antiretroviral pharmacology. Infectious Disease Clinics. 2019;33(3):787-805.

10. Masenyetse LJ, Manda SOM, Mwambi HG. An assessment of adverse drug reactions among HIV positive patients receiving antiretroviral treatment in South Africa. AIDS Research and Therapy. 2015;12(1):6.

11. Pourcher V, Gourmelen J, Bureau I, Bouee S. Comorbidities in people living with HIV: An epidemiologic and economic analysis using a claims database in France. PLoS One. 2020;15(12):e0243529.

12. Edwards IR, Aronson JK. Adverse drug reactions: definitions, diagnosis, and management. The lancet. 2000;356(9237):1255-9.

13. Coleman JJ, Pontefract SK. Adverse drug reactions. Clin Med (Lond). 2016;16(5):481-5.

14. Stover J, Glaubius R, Teng Y, Kelly S, Brown T, Hallett TB, et al. Modeling the epidemiological impact of the UNAIDS 2025 targets to end AIDS as a public health threat by 2030. PLoS medicine. 2021;18(10):e1003831.

15. Ramadhani HO, Thielman NM, Landman KZ, Ndosi EM, Gao F, Kirchherr JL, et al. Predictors of incomplete adherence, virologic failure, and antiviral drug resistance among HIV-

infected adults receiving antiretroviral therapy in Tanzania. Clinical Infectious Diseases. 2007;45(11):1492-8.

16. Montessori V, Press N, Harris M, Akagi L, Montaner JS. Adverse effects of antiretroviral therapy for HIV infection. Cmaj. 2004;170(2):229-38.

17. Kassem LM, Alhabib B, Alzunaydi K, Farooqui M. Understanding Patient Needs Regarding Adverse Drug Reaction Reporting Smartphone Applications: A Qualitative Insight from Saudi Arabia. Int J Environ Res Public Health. 2021;18(8).

18. Srisuriyachanchai W, Cox AR, Jarernsiripornkul N. Exploring Healthcare Professionals' Practices and Attitudes towards Monitoring and Reporting of Severe Adverse Drug Reactions. Healthcare [Internet]. 2022; 10(6).

19. Alqahtani SS, Ahmad S, Alam N, Kashan Syed N, Syed MH, Khardali A, et al. Healthcare professionals' awareness, attitudes and practices towards pharmacovigilance and spontaneous adverse drug reaction reporting in Jazan Province, Saudi Arabia: A survey study. Saudi Pharm J. 2023;31(6):979-88.

20. Shahi A, Shrestha S, K. C B, Acharya K, Pradhan SK. Potential roles of pharmacists in HIV/AIDS care delivery in Nepal: A qualitative study. Plos one. 2023;18(1):e0280160.

21. Burgess LH, Kramer J, Castelein C, Parra JM, Timmons V, Pickens S, et al. Pharmacy-Led Medication Reconciliation Program Reduces Adverse Drug Events and Improves Satisfaction in a Community Hospital. HCA Healthc J Med. 2021;2(6):411-21.

22. Masuka JT, Chipangura P, Nyambayo PP, Stergachis A, Khoza S. A comparison of adverse drug reaction profiles in patients on antiretroviral and antitubercular treatment in Zimbabwe. Clinical drug investigation. 2018;38:9-17.

23. Minzi O, Irunde H, Moshiro C. HIV patients presenting common adverse drug events caused by highly active antiretroviral therapy in Tanzania. Tanzania journal of Health research. 2009;11(1).

24. Yadesa TM, Kitutu FE, Tamukong R, Alele PE. Prevalence, incidence, and characteristics of adverse drug reactions among older adults hospitalized at Mbarara regional referral hospital, Uganda: a prospective cohort study. Clinical Interventions in Aging. 2021:1705-21.

25. Fisher LD. Self-designing clinical trials. Stat Med. 1998;17(14):1551-62.

26. Moirana EL. The Prevalence of Antiretroviral-Therapy-related Adverse Reactions, Hospitalisation, and Mortality among People Living with HIV in Africa-A systematic review and Meta-Analysis: Faculty of Health Sciences; 2022.

27. Ministry of Health U. Uganda Clinical Guidelines 2023: MOH; 2023 [Available from: https://www.health.go.ug/cause/uganda-clinical-guidelines-2023/.

28. Naranjo CA, Busto U, Sellers EM, Sandor P, Ruiz I, Roberts E, et al. A method for estimating the probability of adverse drug reactions. Clinical Pharmacology & Therapeutics. 1981;30(2):239-45.

29. Hartwig SC, Siegel J, Schneider PJ. Preventability and severity assessment in reporting adverse drug reactions. American journal of hospital pharmacy. 1992;49(9):2229-32.

30. Schumock G, Thornton J. Focusing on the preventability of adverse drug reactions. Hospital pharmacy. 1992;27(6):538-.

31. Abah IO, Ncube NBQ, Bradley HA, AgbaJi OO, Kanki P. Antiretroviral Therapy-associated Adverse Drug Reactions and their Effects on Virologic Failure- A Retrospective Cohort Study in Nigeria. Curr HIV Res. 2018;16(6):436-46.

32. Menza M. The Incidence of Adverse Drug Reaction Among Adult Patients on Antiretroviral Therapy in Ethiopia: Frailty Model. HIV AIDS (Auckl). 2022;14:155-65.

33. Jacob J, Radhakrishnan V. A Cross-Sectional Study of Adverse Drug Reaction in Hiv Patients Taking Antiretroviral Treatment from Government Medical College, Kozhikode.

34. Lartey M, Asante-Quashie A, Essel A, Kenu E, Ganu V, Neequaye A. Adverse drug reactions to antiretroviral therapy during the early art period at a tertiary hospital in Ghana. Pan Afr Med J. 2014;18:25.

35. Papa N, Cammarota S, Citarella A, Atripaldi L, Bernardi FF, Fogliasecca M, et al. Evolution in Real-World Therapeutic Strategies for HIV Treatment: A Retrospective Study in Southern Italy, 2014-2020. J Clin Med. 2021;11(1).

36. Ghosh AK. Four decades of continuing innovations in the development of antiretroviral therapy for HIV/AIDS: Progress to date and future challenges. Glob Health Med. 2023;5(4):194-8.

37. Chauhan NS, Shah SP, Desai MK, Shah A. A safety analysis of different drug regimens used in human immunodeficiency virus-positive patients. Indian J Sex Transm Dis AIDS. 2018;39(2):84-90.

38. Mendes JC, Bonolo PF, Ceccato M, Costa JO, Reis AMM, Dos Santos H, et al. Adverse reactions associated with first-line regimens in patient initiating antiretroviral therapy. Eur J Clin Pharmacol. 2018;74(8):1077-88.

39. Mendes JC, Bonolo PdF, Ceccato MdGB, Costa JdO, Reis AMM, Dos Santos H, et al. Adverse reactions associated with first-line regimens in patient initiating antiretroviral therapy. European journal of clinical pharmacology. 2018;74:1077-88.

40. E Koochak H, Babaii A, Pourdast A, Golrokhy R, Rasoolinejad M, Khodaei S, et al. Prevalence of adverse drug reactions to highly active antiretroviral therapy (HAART) among HIV positive patients in Imam Khomeini Hospital of Tehran, Iran. Infectious Disorders-Drug Targets (Formerly Current Drug Targets-Infectious Disorders). 2017;17(2):116-9.

41. Madden VJ, Parker R, Goodin BR. Chronic pain in people with HIV: a common comorbidity and threat to quality of life. Pain Manag. 2020;10(4):253-60.

42. Slawek DE. People living with HIV and the emerging field of chronic pain-what is known about epidemiology, etiology, and management. Curr HIV/AIDS Rep. 2021;18(5):436-42.

43. Sarfo FS, Sarfo MA, Norman B, Phillips R, Chadwick D. Incidence and determinants of nevirapine and efavirenz-related skin rashes in West Africans: nevirapine's epitaph? PLoS One. 2014;9(4):e94854.

44. Neuman MG, Schneider M, Nanau RM, Parry C. HIV-Antiretroviral Therapy Induced Liver, Gastrointestinal, and Pancreatic Injury. Int J Hepatol. 2012;2012:760706.

45. Kiguba R, Zakumumpa H, Ndagije HB, Mwebaza N, Ssenyonga R, Tregunno P, et al. Facilitators and Barriers to Uptake of the Med Safety Mobile App for Adverse Drug Reaction Reporting by Health Workers in Uganda: A Qualitative Study. Drug Saf. 2023;46(6):565-74.

46. Gelaw YK, Adugna B, Tsegaye AT, Melaku T, Kefale B. Coping Strategies for Adverse Effects of Antiretroviral Therapy among Adult HIV Patients Attending University of Gondar Referral Hospital, Gondar, Northwest Ethiopia: A Cross-Sectional Study. AIDS Res Treat. 2018;2018:1879198.

47. Kyajja R, Muliira JK, Ayebare E. Personal coping strategies for managing the side effects of antiretroviral therapy among patients at an HIV/AIDS clinic in Uganda. African Journal of AIDS Research. 2010;9(3):205-11.

48. Bassi PU, Osakwe AI, Ogar CK, Elagbaje C, Nwankwo BB, Balogun ST, et al. Impact of comorbidity on adverse drug reaction profile in a cohort of patients treated with Artemisinin combination therapies for uncomplicated malaria in Nigeria. Pharmacol Res Perspect. 2017;5(2):e00302.

49. Osanlou R, Walker L, Hughes DA, Burnside G, Pirmohamed M. Adverse drug reactions, multimorbidity and polypharmacy: a prospective analysis of 1 month of medical admissions. BMJ Open. 2022;12(7):e055551.

50. Ciccullo A, Baldin G, Borghi V, Lagi F, Latini A, d'Ettorre G, et al. Real-Life Impact of Drug Toxicity on Dolutegravir Tolerability: Clinical Practice Data from a Multicenter Italian Cohort. Viruses. 2022;14(1).

51. Chawla A, Wang C, Patton C, Murray M, Punekar Y, de Ruiter A, et al. A Review of Long-Term Toxicity of Antiretroviral Treatment Regimens and Implications for an Aging Population. Infect Dis Ther. 2018;7(2):183-95.

52. Kommu S, Carter C, Whitfield P. Adverse Drug Reactions. StatPearls. Treasure Island (FL): StatPearls Publishing

Copyright © 2024, StatPearls Publishing LLC.; 2024.

53. Keche Y, Gaikwad N, Dhaneria S. Preventability, predictability, severity and causality assessment of adverse drug reactions reported from a teaching hospital in chhattisgarh: A retrospective analysis. Journal of Family Medicine and Primary Care. 2021;10(7).

54. Kindie E, Alamrew Anteneh Z, Worku E. Time to development of adverse drug reactions and associated factors among adult HIV positive patients on antiretroviral treatment in Bahir Dar City, Northwest Ethiopia. PLOS ONE. 2017;12(12):e0189322.

55. Obiako O, Muktar H, Garko S, Tobi-Ajayi E, Olayinka A, Iyanda M. Adverse reactions associated with antiretroviral regimens in adult patients of a university teaching hospital HIV program in Zaria, Northern Nigeria: An observational cohort study. J Antivir Antiretrovir. 2012;4(9).

56. Sood A, Prajapati H, Bhagra S, Bansal R. Characterization and comparative analysis of ADRs of various ART regimens: experience of our medical college from Western Himalayan region. Int J Res Med Sci. 2017;5(2):659-65.

57. Silva M, Lima MCL, Dourado C, Andrade MS. Aspects related to health literacy, self-care and compliance with treatment of people living with HIV. Rev Esc Enferm USP. 2022;56:e20220120.

58. Tatiparthi RR, Mamo Y. Prevalence of ADRs and associated factors of antiretroviral treatment on HIV positive adults at Jush. Indian Journal of Pharmacy Practice. 2014;7(4).

59. Menza M. The Incidence of Adverse Drug Reaction Among Adult Patients on Antiretroviral Therapy in Ethiopia: Frailty Model. HIV/AIDS-Research and Palliative Care. 2022:155-65.

60. Shelburne SA, Visnegarwala F, Darcourt J, Graviss EA, Giordano TP, White AC, Jr., et al. Incidence and risk factors for immune reconstitution inflammatory syndrome during highly active antiretroviral therapy. Aids. 2005;19(4):399-406.

61. Mataramvura H, Bunders MJ, Duri K. Human immunodeficiency virus and antiretroviral therapy-mediated immune cell metabolic dysregulation in children born to HIV-infected women: potential clinical implications. Frontiers in Immunology. 2023;14.

62. Jiang H, Lin Y, Ren W, Fang Z, Liu Y, Tan X, et al. Adverse drug reactions and correlations with drug–drug interactions: A retrospective study of reports from 2011 to 2020. Frontiers in Pharmacology. 2022;13.

63. Magro L, Moretti U, Leone R. Epidemiology and characteristics of adverse drug reactions caused by drug-drug interactions. Expert Opin Drug Saf. 2012;11(1):83-94.
