# Supplementary material for: Prevalence and factors associated with adverse drug reactions among patients on highly active antiretroviral therapy at a tertiary hospital in south western Uganda: A cross-sectional study
Source: PLoS One. 2025 Apr 10;20(4):e0321015. doi: 10.1371/journal.pone.0321015 (PMC11984723; doi:10.1371/journal.pone.0321015)
Supplement: Attachment — Submitted filename: Response to Reviewers.docx [file pone.0321015.s004.docx]

**Response to Reviewers**

| **Reviewer** | **Comment** | **Response** |
| --- | --- | --- |
| **Reviewer 1** | 1. What was your definition of ADR. It would be better to cite it in the method part | The definition has been included and cited (**Page 6**) |
|  | 2. Since patients were taking a combination of HAART, how do you compute an ADR with a specific medication? | The reported ADR by the participants were evaluated against known adverse reaction profile of each drug using Ugandan Clinical Guidelines 2023 and British National Formulary (BNP) version 85; This was done in order to attribute the reported ADR to specific medication. (**Page 6**) |
|  | 3. In the method section, it would be better if you could explain how, you differentiated the ADRs from a disease symptom or if the ADR happens from an ADR of another medication the patient was taking at that point | Naranjo ADR assessment scale was used which is a standard tool used determine the causal relationship. The scale helps to determine if the adverse reaction observed is due to the suspected drug or another source be it a disease or other medication (**Page 6**) |
|  | 4. Table 4: Commonly Reported ADRs among patients with HIV on HAART at MRRH (doesn’t have the frequency) | Frequency has been included in the table |
|  | 5. Most of the ADRs look like a subject (patient) reported and it cannot be confirmed. So how did you judge if it is an ADR. | The Naranjo ADR assessment scale is comprehensive, a standard questionnaire which assigns probability scores, it among other things examines whether the ADR reported started before the initiation of the drug or not. This does not necessarily confirm the ADR but rather provides a probability score in the range of “Doubtful – Possible – Probable – Definite”. As reported under the result section (**Pages 13, 14 & Figure 3**). The limitation of a possible recall bias due to patient reports was acknowledged under “Limitations of the study” section (**Page 19**) |
|  | 6. How did you characterize some of the ADRs like ‘difficulty of sleeping’ … how many hours of sleep or how do you define this ADR? The same works for Low appetite, diarrhea, excessive eating, drinking…… and so on. (Better to define them in the methods) | Since ADRs were not predetermined they could not be defined in advance under methods; although all ADRs identified were assessed and reported based on their standard definitions and assessment criteria. Some of the ADRs have been defined under Table 4 (**Page 15**) |
|  | 7. Since you only had a onetime interaction with the patients (Cross-sectional study), How did you determine the renal problems? Was it based on the data they had in the past? | Objective data on renal function tests was obtained from the reviewed patient charts. An abnormal renal function test result was assessed based on Naranjo scale and known ADR profile of the HAART drugs to determine the causal relationship. |
|  | 8. The Discussion could comment more on implications of findings for practice and policy, like what the different stakeholders can do to improve the situation. | The discussion section has been improved to include specific recommendations for different stakeholders. More recommendations are summarized under the “Conclusion section”. |
| **Reviewer 2** | The HAART was defined, but the auditors did not indicate the adherence level of these Persons living with HIV | This has been acknowledged under limitations of the study |
|  | Also, they did not account for other medications that PLWHIV could be on, be it multivitamins or prophylaxis for Opportunistic infections for instance | This was put into account as indicated in Table 5: Other medications including OTCs and herbal medicines use, were evaluated for association with development of ADR |
| **Reviewer 3** | There are some corrections which I have indicated in the attached file for the authors to look at. | The comments have been attended to accordingly throughout the document. |
