# Supplementary material for: Prevalence and factors associated with adverse drug reactions among patients on highly active antiretroviral therapy at a tertiary hospital in south western Uganda: A cross-sectional study
Source: PLoS One. 2025 Apr 10;20(4):e0321015. doi: 10.1371/journal.pone.0321015 (PMC11984723; doi:10.1371/journal.pone.0321015)
Supplement: Attachment — Submitted filename: Response_to_Reviewers_auresp_2.docx [file pone.0321015.s005.docx]

**Response to Reviewers**

| **Reviewer** | **Comment** | **Response** |
| --- | --- | --- |
| **Reviewer 3** | I raised quite a number of concerns in the first review. However, NONE of the concerns raised were addressed. I think the authors will have to take a look at it again. I did attach a file in PDF with sticky notes on what corrections needs to be addressed. I have therefore attached it again for the concerns to be addressed. | Thank you very much for your crucial comments. The authors believe your comments have helped us to significantly improve our write-up. We have now addressed all the raised comments throughout the document |
